# Supplementary material for: Empirical methods for controlling false positives and estimating confidence in ChIP-Seq peaks
Source: BMC Bioinformatics. 2008 Dec 5;9:523. doi: 10.1186/1471-2105-9-523 (PMC2628906; doi:10.1186/1471-2105-9-523)
Supplement: Additional File 1 — A variety of html documents from the USeq web site detailing the available applications, their best usage, output file type descriptions, command line menus, etc. [file 1471-2105-9-523-S1.zip › USeqUserGuides/JavaDoc.zip!/JavaDoc/trans/graphics/Graph.html]

Graph


|  |  |  |
| --- | --- | --- |
| **Overview** \| **Package** \| **Class** \| **Use** \| **Tree** \| **Deprecated** \| **Index** \| **Help** | |  |
| **PREV CLASS**   **NEXT CLASS** | **FRAMES**    **NO FRAMES**     **All Classes** |  |
| SUMMARY: NESTED \| FIELD \| CONSTR \| METHOD | DETAIL: FIELD \| CONSTR \| METHOD |  |


---


## trans.graphics Class Graph

```
java.lang.Object
  trans.graphics.Graph
```

---

``` public class Graph extends java.lang.Object ```

Graph for `IntervalPlotter`.

---

**Constructor Summary**

`Graph(double[] values,
double[] positions,
double width,
double height,
java.awt.Color dataLineColor)`


| **Method Summary** | |
| --- | --- |
| `void` | `draw(java.awt.Graphics2D g2, java.awt.font.FontRenderContext fontContext, java.awt.Font font)` |
| `double` | `getPixelsPerBase()` |
| `double[]` | `getPositions()` |
| `double` | `getWOffSet()` |
| `double` | `getX()` |
| `void` | `makeDataLines()` |
| `double` | `makeGlyphs(double topLeftXCoor, double topLeftYCoor, double widthScalar, double heigthScalar)`             Returns bottom y coordinate of graph. |
| `void` | `makeGridLines()` |
| `void` | `scaleValuesPositions()` |
| `void` | `setMinMaxY(double[] minMaxY)`             Use to set a common y scale, pass it a minMax for the entire dataset. |

**Methods inherited from class java.lang.Object**

`equals, getClass, hashCode, notify, notifyAll, toString, wait, wait, wait`

**Constructor Detail**

### Graph

```
public Graph(double[] values,
             double[] positions,
             double width,
             double height,
             java.awt.Color dataLineColor)
```


**Method Detail**

### setMinMaxY

```
public void setMinMaxY(double[] minMaxY)
```

:   Use to set a common y scale, pass it a minMax for the entire dataset.

---


### makeGlyphs

```
public double makeGlyphs(double topLeftXCoor,
                         double topLeftYCoor,
                         double widthScalar,
                         double heigthScalar)
```

:   Returns bottom y coordinate of graph.

---


### draw

```
public void draw(java.awt.Graphics2D g2,
                 java.awt.font.FontRenderContext fontContext,
                 java.awt.Font font)
```

---


### makeGridLines

```
public void makeGridLines()
```

---


### scaleValuesPositions

```
public void scaleValuesPositions()
```

---


### makeDataLines

```
public void makeDataLines()
```

---


### getWOffSet

```
public double getWOffSet()
```

---


### getX

```
public double getX()
```

---


### getPixelsPerBase

```
public double getPixelsPerBase()
```

---


### getPositions

```
public double[] getPositions()
```


---


|  |  |  |
| --- | --- | --- |
| **Overview** \| **Package** \| **Class** \| **Use** \| **Tree** \| **Deprecated** \| **Index** \| **Help** | |  |
| **PREV CLASS**   **NEXT CLASS** | **FRAMES**    **NO FRAMES**     **All Classes** |  |
| SUMMARY: NESTED \| FIELD \| CONSTR \| METHOD | DETAIL: FIELD \| CONSTR \| METHOD |  |


---
